# Effects of dietary vinegar on performance, immune response and small intestine histomorphology in 1‐ to 28‐day broiler chickens

**DOI:** 10.1002/vms3.408

**Published:** 2020-12-16

**Authors:** Mohammad Jahantigh, Heydar Kalantari, Seyedeh Ayda Davari, Dariush Saadati

**Affiliations:** ^1^ Department of Clinical Sciences School of Veterinary Medicine University of Zabol Zabol Iran; ^2^ Department of Pathobiology School of Veterinary Medicine University of Zabol Zabol Iran; ^3^ Department of Food Hygiene School of Veterinary Medicine University of Zabol Zabol Iran

**Keywords:** Broilers, Immune response, Intestinal histomorphology, Performance, Vinegar

## Abstract

The purpose of this research was to evaluate the effects of apple cider vinegar in diet on the growth performance, immune response, histomorphological changes of the small intestine and some serum biochemical factors in broilers. A total of 85 broiler chicks of Ross 308 were purchased and 64 well‐conditioned chicks were selected and divided into four experimental groups with four replicates and four chicks per replicate. The diets of groups 2, 3 and 4 were mixed with 1, 2 and 3% of vinegar, respectively, and group 1 as the control group was fed by the standard diet. The results showed that body weight gain was higher in the groups fed vinegar than the control group. There were no significant changes in the feed conversion ratio between the treatment and control groups (*p* = .507). Vinegar intake through the diet did not change significantly the weight of Bursa of Fabricius (*p* = .369) and spleen (*p* = .122). Vinegar significantly reduced blood urea nitrogen levels in the treatment groups compared with the control group (*p* = .0052). There was a significant increase in the level of antibody titre against Newcastle disease virus by haemagglutination inhibition test in the groups receiving vinegar in comparison with control group (*p* = .0358). Compared with the control group, the villus height (*p* = .0022) and intestinal crypts depth (*p* = .0015) significantly increased in the groups receiving apple cider vinegar. In conclusion, dietary supplementation with apple cider vinegar has beneficial effects on performance, immune response and small intestine histomorphology in broilers.

## Graphical abstract

The purpose of this research was to evaluate the effects of apple cider vinegar in diet on the growth performance, immune response, histomorphological changes of the small intestine and some serum biochemical factors in broilers. According to the results, dietary supplementation with apple cider vinegar has beneficial effects on performance, immune response and small intestine histomorphology in broilers.

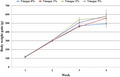

## 1 INTRODUCTION

Recently, efforts have been made to reduce environmental contamination and produce high‐quality animal products by efficient utilization of natural substances. The use of acidifier could be potentially useful in the poultry performance improvement (Attia et al., 2013; Tasharofi et al., 2017). Vinegar is a liquid that contains about 5%–20% of acetic acid. Vinegar also contains chemicals such as: anthocyanins, flavanols, amino acids, vitamins, mineral salts, non‐volatile acids, polyphenolic compounds and water (Okoye & Porolo, 2019). Apple vinegar has antibacterial and antifungal properties (Shahidi et al., 2008). Hamilton et al. (2020) observed that apple cider vinegar had no effect on oocyst shedding of *Eimeria* spp. in chickens (Hamilton et al., 2020). Hayajneh et al. (2018) reported the antioxidative role of apple cider vinegar in infected chickens (Hayajneh et al., 2018). Beta‐carotene in apple cider vinegar has antioxidant properties. Consumption of apple cider vinegar improves the immune response against pathogens and also contributes to the acid–base balance (Shahidi et al., 2008). The main chemical compound of vinegar is organic acids such as acetic acid (Tasharofi et al., 2017). The most research related to vinegar emphasizes on the effect of organic acids like acetic acid (Allahdo et al., 2018; Tasharofi et al., 2017). Organic acids are added to feed for their various beneficial effects on gut function and improving immune response (Allahdo et al., 2018; Attia et al., 2013). The best time to consume organic acid is the initial period of broiler breeding (Leeson et al., 2005). Previous studies have shown that the use of organic foods and organic acids increases the height of intestinal villi in poultry (Abdel‐Fattah et al., 2008; Baurhoo et al., 2007; Xu et al., 2003). Increasing the height of intestinal villi increases the absorption of nutrients in the small intestine and subsequently increases weight and improves the performance of chickens (Awad et al., 2009). The consumption of vinegar through feed at the early ages is more useful and can also be economical. This study has a new insight on the effect of using natural apple cider vinegar in broilers. So far, most research on the use of vinegar in broilers has been in drinking water. This research was designed to study the effect of locally produced apple cider vinegar in the diets on the growth performance, immune system, some serum biochemical factors and histomorphological changes of the small intestine in broilers from 1 to 28 days.

## 2 MATERIALS AND METHODS

### 2.1 Birds, housing, diet, vinegar

To do this study, 85 one‐day‐old unsexed broiler chicks of Ross 308 were purchased. Sixty‐four chickens with the better condition were selected and then chicks were divided randomly into four experimental groups with four replicates and four chicks per replicate. Before arrival of chicks the room cleaned and disinfected and divided into 16 pens. Each pen has one square metre space and, its floor covered with wood shaving. The initially temperature at the entrance days of the chickens was 32°C and gradually decreased about 0.5°C every day to reached the constant temperature of 20–22°C. The exposure programme was 24‐hr light during the first 2 days of chick‐rearing and 23‐hr light and 1‐hr dark from the third day until the end of the experiment. The present study was performed in Zabol, north of Sistan and Baluchestan province, Iran and the Ethics Committee of University of Zabol, approved the experimental procedure (Ethics no: IR.UOZ.REC.1395.009).

Chickens' diets were prepared and used according to the standard dietary requirements based on corn and soybean (Table 1). The basal diets were formulated to provide the nutritional requirements of the chicks. During the experiment, the chicks were given free access to water and food in mash form. The diets of chickens of groups 1, 2, 3 and 4 were mixed by spraying with 0, 1, 2 and 3% of apple vinegar, respectively. The amount of apple cider vinegar in the diets was selected on the basis of previous studies (Tasharofi et al., 2017). The supplemented diet of each group with mentioned percentage of vinegar was freshly prepared and used from 1 to 28 days of the experiment. For this purpose 1 kg of feed was mixed with 10, 20 and 30 ml of vinegar to prepare the diets with 1%, 2% and 3% of vinegar, respectively.

**TABLE 1:** Feed ingredients and calculated nutrient composition of the experimental basal diets of chicks during 1–28 days of age

Ingredient (g/kg)	Starter (1–14 days of age)	Grower (15–28 days of age)
Corn	622	658
Soybean meal, 44% CP	293	269
Fish meal	60	45
Dicalcium phosphate	9	12
Oyster	8	8
Sodium chloride	1	1.5
Vitamin premixes^1^	2.50	2.50
Mineral premixes^2^	2.50	2.50
DL‐Methionine, 98%	1.4	1.1
L‐Lysine HCl, 98%	0.6	0.4
Calculated nutrient composition		
Metabolizable energy, kcal/kg	2,937	2,958
Crude protein, %	22.19	20.50
Calcium, %	0.95	0.93
Available phosphorus, %	0.48	0.49
Methionine, %	0.55	0.48
Lysine, %	1.33	1.18
Methionine + cystein, %	0.91	0.82

Note

1‐Provides (per kg of diet): vitamin A, 9,000 IU; vitamin D3, 2000 IU; vitamin E, 18 mg; vitamin k3, 2 mg; thiamin, 1.8 mg; riboflavin, 6.6 mg; niacin, 10 mg; pantothenic acid, 30 mg; pyridoxine, 3 mg; folic acid, 1 mg; vitamin B12, 0.015 mg; biotin, 0.1 mg; choline chloride, 250 mg, and antioxidant, 1.0 mg.

2‐Provides (per kg of diet): manganese, 100 mg; zinc, 85 mg; iron, 50 mg; copper, 10 mg; iodine, 1 mg; selenium, 0.2 mg, and choline chloride 250 mg.

To prepare vinegar, apple fruits were sliced and their seeds removed and then placed in a jar. Diluted industrial vinegar (1:4 water and vinegar) were added and the jar transferred to a warm and dark place for 3 weeks. Finally, the solid parts removed and the liquid used as apple cider vinegar.

**Figure 1:**
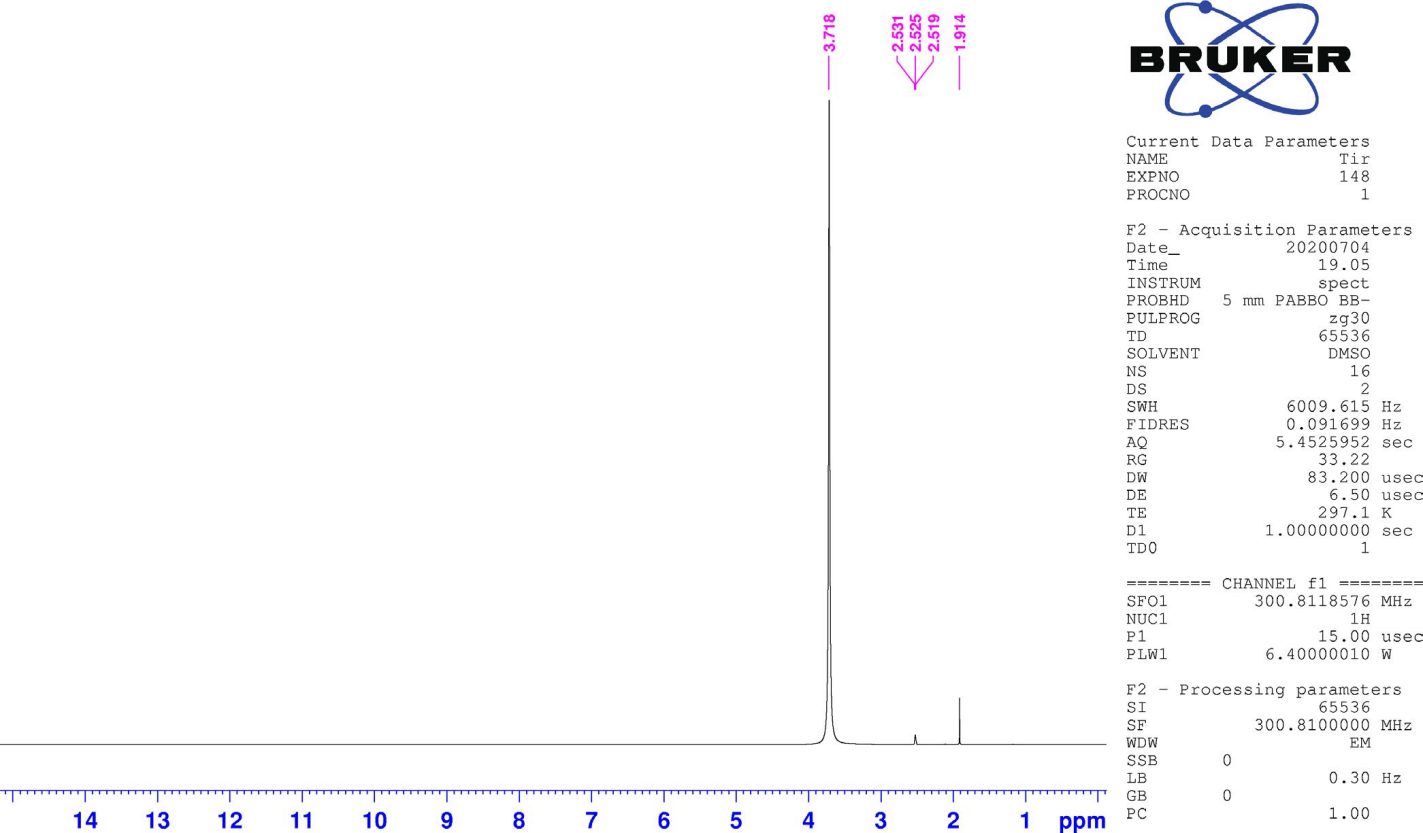
1H NMR spectrum of the apple cider vinegar is presented

Proton nuclear magnetic resonance (^1^H NMR) spectroscopy was performed to confirm the existence of acetic acid in the vinegar liquid. As shown in Figure 1, 1H NMR spectrum confirmed the presence of the acetic acid by the distinctive feature of the methyl signals at *δ = *1.9 (as a singlet).

Feed intake of chicks was recorded and the feed conversion ratio was calculated for 28 days. All chicks were vaccinated by spraying at 1 day of age with Newcastle disease B1 vaccine and at 8 days of age with Newcastle oil emulsion vaccine (Razi Vaccine and Serum Research Institute, Iran).

### 2.2 Sampling and jejunum histomorphology

On days 14 and 28, a chick from each replicate was randomly selected, weighed and followed by blood sampling, anaesthetized first and then was exposed to necropsy. After the necropsy, the organs of the Bursa of Fabricius, spleen and entire small intestine were removed and weighed with contents. The length of the small intestine from the beginning of the duodenum to the location of the blind intestines was also measured. For histomorphological changes of the small intestine, the tissue specimen was prepared with a dimension of 1 × 1 × 0.5 cm from the jejunum part and fixed in 10% neutral buffered formalin. Tissues were dehydrated by transferring through series of alcohols with increasing concentrations, placed into xylene and embedded in paraffin. A microtome was used to make some sections that were of 5 μm. The paraffin sections were stained with haematoxylin‐eosin and examined by the light microscopy under different magnifications (Tasharofi et al., 2017). Similar to the method of Houshmand et al. (2012), the factors of four intestinal villi including villus height, villus width, crypt depth and crypt depth to villus height ratio were measured by micrometry in each chick and the mean values were reported as the chicken intestinal villus size (Houshmand et al., 2012).

### 2.3 Serum Biochemical analysis, HI test

Blood samples were centrifuged at 500 g for 5 min, and sera were collected and stored at −20°C until use. Serum samples were used for the determination of cholesterol, triglyceride, albumin, total protein, blood urea nitrogen (BUN), low density lipoprotein (LDL), high density lipoprotein (HDL) and haemagglutination inhibition (HI) antibody titre. Blood serum biochemical factors were measured by an Auto Analyzer Selectra Pro *M* (Netherlands). HI test was used to measure serum antibody titre against Newcastle disease virus. The HI titre was expressed as the log_2_ reciprocal of the highest serum dilution producing 100% inhibition of HA activity (Rahimi & Khaksefidi, 2006).

### 2.4 Statistical analysis

Data were subjected to analysis of variance (ANOVA) procedure of SPSS Software version 25 (SPSS Inc., Chicago, IL, United States). Replicates were considered as the experiment units. The statistical model used was: Yij = μ+Ti + Eij, where: Yij = response variables from each experimental unit; μ = the overall mean; Ti = i‐th treatment effect; Eij = the random terms of study. Multiple pairwise comparisons of means were performed by Duncan's post hoc test. Due to the graded level of vinegar, orthogonal polynomial contrasts were used to reveal the linear and quadratic dose response associations. The significance level was considered to be *p* < .05 for the main effects and *p* < .1 for linear and quadratic trends. Results were reported as mean ± standard deviation.

## 3 RESULTS

**Figure 2:**
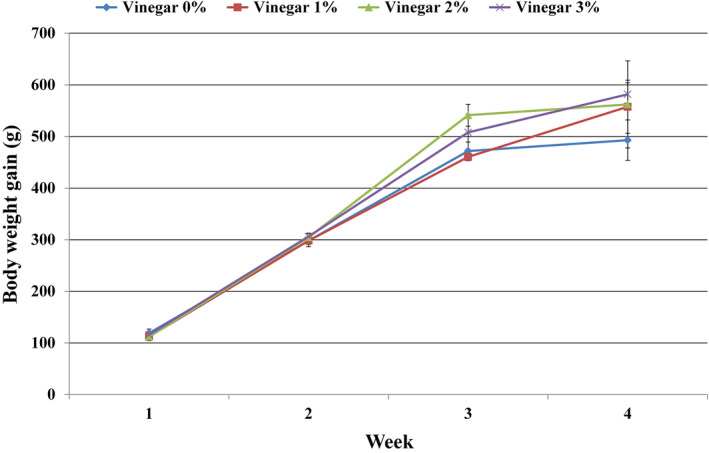
Chicks weight gain in the control and treatment groups at different weeks of experiment has been shown

The values measured for weight gain during the different weeks of testing in the control and treatment groups have been shown in Figure 2. As shown in Figure 2, body weight gain was higher in the groups fed by apple cider vinegar than the control group.

Results for feed conversion (FC) ratio, the internal organ weights including small intestine, the weight of Bursa of Fabricius and spleen and small intestine length in the control and treatment groups have been shown in Table 2. According to Table 2, the use of vinegar in the diet does not significantly change the FC ratio for 1–28 days (*p* = .507). As shown in Table 2, the vinegar intake through the diet did not cause significant changes in the weight of Bursa of Fabricius (*p* = .369) and spleen (*p* = .122), but vinegar intake increased the weight gain of the Bursa of Fabricius, although this increase is not significant.

**TABLE 2:** Feed conversion, weight of internal organs including small intestine, the weight of Bursa of Fabricius and spleen, small intestine length and the ratio of the weight of internal organs to the body weight in the different groups on day 28 of the experiment

Organ	Vinegar 0%	Vinegar 1%	Vinegar 2%	Vinegar 3%	Contrast *p* value	
					Linear	Quadratic
FC	1.32 ± 0.181**^a^**	1.44 ± 0.106**^a^**	1.36 ± 0.567**^a^**	1.43 ± 0.143**^a^**	0.387	0.687
Int Wt (g)	71.5 ± 3.49**^a^**	84.3 ± 14.0**^a^**	71.2 ± 17.3**^a^**	75.8 ± 7.91**^a^**	0.996	0.503
BF Wt (g)	2.39 ± 0.236**^a^**	3.39 ± 0.760**^a^**	3.29 ± 1.50**^a^**	3.20 ± 0.282**^a^**	0.247	0.226
Sp Wt (g)	1.18 ± 0.376**^a^**	1.50 ± 0.262**^a^**	1.58 ± 0.200**^a^**	1.24 ± 0.0716**^a^**	0.654	0.0227
BF Wt/ B Wt(%)	0.167 ± 0.0188**^a^**	0.213 ± 0.0544**^a^**	0.199 ± 0.0740**^a^**	0.196 ± 0.0242**^a^**	0.510	0.328
Sp Wt/ B Wt(%)	0.0822 ± 0.0252**^a^**	0.0946 ± 0.0230**^a^**	0.0975 ± 0.0110**^a^**	0.0759 ± 0.0082**^a^**	0.705	0.090
Int Wt/ B Wt (%)	5.00 ± 0.257**^a^**	5.27 ± 0.983**^a^**	4.40 ± 0.901**^a^**	4.62 ± 0.294**^a^**	0.221	0.942
Int leng (mm)	158 ± 6.91**^a^**	169 ± 11.4**^a^**	165 ± 23.8**^a^**	176 ± 8.26**^a^**	0.149	0.966

Abbreviations FC, feed conversion ratio for 1–28 days; Int, intestine; Sp, spleen; Leng, length; Wt, weight; B, body; BF, Bursa of Fabricius.

* The values in each row with non‐identical English letters have a significant difference.

There was no observed statistical significant difference between control and treatment groups at 14 days, regarding the weights of the internal organs (statistical results not shown).

The measured values for the biochemical factors of blood serum and HI antibody titre have been given in Table 3. As shown in Table 3, vinegar reduced blood serum BUN levels, so that 2% and 3% of it, decreases BUN significantly compared with the control group (*p* = .0052). There was no significant difference between control and treatment groups in the levels of cholesterol, albumin, total protein, triglyceride, HDL and LDL of blood serum. However, apple cider vinegar has reduced HDL levels in blood serum. As shown in Table 3, there was a significant difference in the antibody titre of HI between the groups receiving apple cider vinegar and the control group (*p* = .0358). Moreover, for BUN and HI titre the main effects and Linear trend effects were statistically significant.

**TABLE 3:** Biochemical factors of blood serum and HI antibody titre between the different groups on day 28 of the experiment

Parameters	Vinegar 0%	Vinegar 1%	Vinegar 2%	Vinegar 3%	Contrast *p* value	
					Linear	Quadratic
BUN (g/dl)	3.25 ± 0.500**^c^**	2.50 ± 0.577**^bc^**	2.25 ± 0.500**^ab^**	1.50 ± 0.577**^a^**	0.0007	1.00
Chol (g/dl)	131 ± 14.0**^a^**	129 ± 10.7**^a^**	133 ± 15.3**^a^**	128 ± 9.75**^a^**	0.802	0.832
HDL (%)	81.0 ± 9.16**^a^**	76.1 ± 9.31**^a^**	77.4 ± 4.10**^a^**	75.1 ± 5.43**^a^**	0.336	0.735
LDL (%)	59.8 ± 10.8**^a^**	58.3 ± 12.8**^a^**	55.3 ± 8.06**^a^**	59.5 ± 6.95**^a^**	0.869	0.573
TG (g/dl)	62.3 ± 16.7**^a^**	69.5 ± 27.7**^a^**	58.8 ± 11.8**^a^**	61.5 ± 8.81**^a^**	0.750	0.805
Alb (g/dl)	1.38 ± 0.0614**^a^**	1.35 ± 0.0545**^a^**	1.40 ± 0.106**^a^**	1.35 ± 0.112**^a^**	0.870	0.758
Total Pr(g/dl)	3.03 ± 0.0519**^a^**	2.84 ± 0.112**^a^**	3.01 ± 0.244**^a^**	3.07 ± 0.536**^a^**	0.666	0.418
HI (log_2_)	4.25 ± 0.500**^a^**	4.25 ± 0.500**^a^**	5.50 ± 0.577**^b^**	5.25 ± 0.957**^ab^**	0.0140	0.712

Abbreviations: BUN, blood urea nitrogen; Chol, cholesterol; HDL, high density lipoprotein; LDL, low density lipoprotein; TG, triglyceride; Alb, albomin; Total Pr, total protein; HI, haemagglutination inhibition.

* The values in each row with non‐identical English letters have a significant difference

The findings of histomorphologic studies of the small intestine including villus width, villus height, crypt depth and the ratio of crypt depth to the villus height have been given in Table 4. As shown in Table 4, there was a significant increase in the villi height (*p* = .0022) and intestinal crypt depth (*p* = .0015) in the groups receiving apple vinegar compared with the control group. Furthermore, the villus height and crypt depth Linear and Quadratic trends were statistically significant.

**TABLE 4:** Histomorphological parameters of the small intestine between the different groups on day 28 of the experiment

Tissue	Vinegar 0%	Vinegar 1%	Vinegar 2%	Vinegar 3%	Contrast *p* value	
					Linear	Quadratic
Villus width (µm)	7.13 ± 1.21**^a^**	9.7 ± 2.09**^a^**	8.13 ± 1.05**^a^**	8.18 ± 0.512**^a^**	0.609	0.0841
Villus height (µm)	65.6 ± 11.4**^a^**	144 ± 29.4**^b^**	126 ± 37.7**^b^**	150 ± 15.7**^b^**	0.0016	0.0521
Crypt depth (µm)	76.6 ± 12.9**^a^**	165 ± 30.5**^b^**	147 ± 42.6**^b^**	170 ± 11.6**^b^**	0.0011	0.0342
CD/VH (%)	1.17 ± 0.0068**^a^**	1.15 ± 0.0241**^a^**	1.17 ± 0.0412**^a^**	1.14 ± 0.0466**^a^**	0.459	0.831

Abbreviations CD, crypt depth; VH, villus height.

* The values in each row with non‐identical English letters have a significant difference

## 4 DISCUSSION

In this study, the chicks receiving apple cider vinegar had a higher weight gain than the control group containing chicks 1‐–28 days of age. Tasharofi et al. (2017) observed that usage of waste date's vinegar increases body weight of broiler chickens (Tasharofi et al., 2017). Vinegar, in addition to acetic acid, contains other nutrients such as vitamins and minerals that can be effective in weight gain (Johnston & Gaas, 2006). Due to low levels of short chain fatty acids in the intestine of young chicks, they may be the best candidates for use of acidifiers (Attia et al., 2013; Panda et al., 2009). As the age increases, the production of volatile fatty acids in the gastrointestinal tract increases; this may be the main reason for the lack of effective addition of organic acids to the feed (Hernandez et al., 2006; Leeson et al., 2005).

According to the results of this research, there was an increase in the factors related to the chickens' immune system—including the weight of Bursa of Fabricius, spleen weight and antibody titre of HI in the groups receiving vinegar via feed compared with the control group. Haque et al. (2010) reported that the addition of organic acid increased the number of cells involved in the immune system in the follicles and increased the weight of the Bursa of Fabricius (Haque et al., 2010). Attia et al. (2013) observed that acetic acid in the diets of *Japanese quail* affected the immune system as manifested by an excess of cellular reactions in the intestine as well as lymphoid hyperplasia in the spleen tissue (Attia et al., 2013). These can be consistent with the results of our research. However, the results of this research are inconsistent with those of Brisbin et al. (2008) who reported no effect of apple vinegar on the weight of lymphoid organs (Brisbin et al., 2008). Besides, Allahdo et al. (2018) observed that vinegar had no significant effect on relative weight of lymphoid organ (Allahdo et al., 2018).

The results of histomorphological studies of intestinal tissue showed that the villus height, as well as intestinal crypt depth, were significantly increased in the treatment groups compared with the control group. Allahdo et al. (2018) also observed that the drinking water supplemented with vinegar significantly increased villus height and crypt depth of small intestine in broilers (Allahdo et al., 2018). In addition, Tasharofi et al. (2017) reported that the intestinal morphology is improved by adding waste date's vinegar to diet of broilers (Tasharofi et al., 2017). The more the villi height, the more the small intestine absorption capacity. The higher villus, inhibits faster passing, reduces the moisture of contents and improves the FC ratio (Awad et al., 2009). Various factors such as the presence of pathogens, different chemicals, stressful conditions, the microflora of the small intestine and the health status of the intestinal epithelium can influence the effect of organic acids and probiotics, on the histopathology of the small intestine (Paul et al., 2007). In the present study, apple cider vinegar significantly increased the height of intestinal villi in the treatment groups compared with the control group, which may be due to the improvement of the intestinal microbial flora status by the use of organic acids (Attia et al., 2013; Baurhoo et al., 2007; Xu et al., 2003).

Significant differences were observed between the control and treatment groups in the serum BUN level. Chickens fed by vinegar through the diet showed a significant decrease in blood BUN levels compared with the control group. There were no significant differences in the levels of cholesterol, triglyceride, albumin, total protein, LDL and HDL between the control and treatment groups. Hayajneh (2019) reported that apple cider vinegar as a natural feed additive has no significant effects on serum total protein, total cholesterol and triglyceride of broiler chickens (Hayajneh, 2019). However, Berrama et al. (2018) reported that apple cider vinegar reduces blood cholesterol and triglyceride levels in broilers (Berrama et al., 2018). Pourmozaffar et al. (2019) also reported that apple cider vinegar reduced cholesterol and triglyceride levels in white shrimp (Pourmozaffar et al., 2019). According to the results of research done by Bouazza et al. (2016), the vinegar has protective effects on the liver and improves liver activity (Bouazza et al., 2016). Attia et al. (2013) and Naziroglu et al. (2014) reported that apple cider vinegar has protective effects on the liver and kidney and reduces serum lipid levels and increased antioxidant enzymes (Attia et al., 2013; Naziroglu et al., 2014). Franca and Treasure (2020) reported that apple cider vinegar ‘with mother’ had no significant effects on kidney function (creatinine and urea) and haematological parameters of Wistar albino rats (Franca & Treasure, 2020).

## 5 CONCLUSION

The study results have shown beneficial effects of dietary apple cider vinegar on growth performance of broiler chickens. The addition of 2% vinegar to the diets of broiler may be used to improve growth, immune response and small intestine histomorphology.

## CONFLICT OF INTEREST

Authors declare that they have no conflict of interest.

## AUTHOR CONTRIBUTION

**Mohammad Jahantigh:** Funding acquisition; Investigation; Methodology; Project administration; Writing‐original draft; Writing‐review & editing. **Heydar Kalantari:** Investigation; Methodology. **Seyedeh Ayda Davari:** Investigation; Methodology; Writing‐original draft. **Dariush Saadati:** Formal analysis; Writing‐original draft; Writing‐review & editing.

## ETHICAL STATEMENT

All the experiments were done in accordance with the principles for care and use of laboratory animals, and the Ethics Committee of the University of Zabol reviewed and approved the protocol (IR.UOZ.REC.1395.009).

### Peer Review

The peer review history for this article is available at https://publons.com/publon/10.1002/vms3.408.
